# Supplementary material for: Mineral surface chemistry control for origin of prebiotic peptides
Source: Nat Commun. 2017 Dec 11;8:2033. doi: 10.1038/s41467-017-02248-y (PMC5725419; doi:10.1038/s41467-017-02248-y)
Supplement: Supplementary file 1 — Supplementary Information [file 41467_2017_2248_MOESM1_ESM.pdf]

Supplementary Table 1: Summary of the systems modelled, their composition and simulation times

**Realistic mix*: relative amino acid concentration matching naturally occurring one[1]. MIX3: 10 dimeric and 15 hexameric different random chains produced with *Assemble!*[2] and replicated in the box. These had amino acid concentration: ALA 26.0%, ASP 18.3%, HIS 7.7%, LYS 15.4%, LEU 22.1%, TYR 10.6%. MIX4: three 24 amino acid-long chains were randomly created with *Assemble!*[2], total concentration of amino acids: ALA 19.4%, ASP 9.7%, HIS 2.8%, LYS 16.7%, LEU 36.1%, TYR 6.9%.

<table>
<tr><th>System name</th><th>LDH, no of unit cells, x y z, total</th><th># of amino acids</th><th>Charge per amino acid/peptide</th><th>Counterbalancing ions</th><th># of water</th><th>Volume, x y z, nm</th><th>Simulation time</th><th>Description</th></tr>
<tr><td>ASP</td><td rowspan="8">15x24x5 total 1800</td><td>180 per layer, total 900</td><td>-2</td><td></td><td rowspan="7">Stepwise: 20, 15, 10, 7, 5, 3, 2 and 0 per AA</td><td rowspan="8">From ~11x11x12 to ~ 11x11x4</td><td rowspan="8">10 ns per dehydration step</td><td rowspan="7">Stepwise dehydration</td></tr>
<tr><td>LYS</td><td>360 per layer, 1800 total</td><td>0</td><td>1800 Cl^-</td></tr>
<tr><td>ALA</td><td rowspan="4">360 per layer 1800 total</td><td rowspan="4">-1</td><td rowspan="4"></td></tr>
<tr><td>LEU</td></tr>
<tr><td>HIS</td></tr>
<tr><td>TYR</td></tr>
<tr><td>MIX1</td><td>60 AA of each type per layer, 1800 total</td><td>As above</td><td></td></tr>
<tr><td>MIX2</td><td>24 AA of each type per layer, 720 total</td><td>As above</td><td>1080 Cl^-</td><td>From 20 to 0 per anion</td><td>Dehydration, 40% amino acid charge balancing load</td></tr>
<tr><td>2ASP</td><td rowspan="6">10x18x1 total 180</td><td>90</td><td>-3</td><td>90 Na^+</td><td rowspan="6">~20 waters per AA ~7000 total</td><td rowspan="6">~7x8x5</td><td rowspan="6">20 ns</td><td rowspan="6">Dipeptide in the hydrated interlayer</td></tr>
<tr><td>2LYS</td><td>180</td><td>+1</td><td>320 Cl^-</td></tr>
<tr><td>2ALA</td><td rowspan="4">180</td><td rowspan="4">-1</td><td></td></tr>
<tr><td>2LEU</td><td></td></tr>
<tr><td>2HIS</td><td></td></tr>
<tr><td>2TYR</td><td></td></tr>
</table>

Supplementary Table 1, contd.

System name	LDH, no of unit cells, *x y z, total*	# of amino acids	Charge per amino acid/peptide	Counterbalancing ions	# of water	Volume, *x y z*, nm	Simulation time	Description
6ASP	10x18x1	90	-7	450 Na^{+}	~20 waters per AA ~20000 total	~7x8x11	20 ns	Hexapeptide in the hydrated interlayer
6LYS		90	+5	630 Cl^{-}				
6ALA		180	-1					
6LEU								
6HIS								
6TYR								
MIX3	10x18x1	15 dimer 15 hexamer see below	See composition below	149 Cl^{-}	~60 water per AA ~8000 total	~7x8x5	50 ns	Realistic mix* of di- and hexa- peptides at 40% of charge balancing load
MIX4	10x18x1	3 x 24mer See below	See composition below	182 Cl^{-}	~100 water per AA ~7200 total	~7x8x4	50 ns	Dilute realistic mix* of three 24mer peptides

Supplementary Table 2

Calculated atomic charges of C-terminal atoms of alanine in vacuum and adsorbed onto the LDH surface with Hirshfeld population analysis.

**System**	**Atomic charge, e**	
	**C**	**O**
1 ALA vacuum	0.06	-0.4, -0.42
4 ALA vacuum	0.09, 0.07, 0.08, 0.07	-0.4, -0.4, -0.4, -0.43, -0.35, -0.4, -0.42, -0.42
**Average**	**0.074 ± 0.011**	**-0.405 ± 0.022**
1 ALA adsorbed on LDH	0.19	-0.22, -0.23
4 ALA adsorbed on LDH	0.17, 0.16, 0.18, 0.18	-0.23, -0.26, -0.28, -0.21, -0.23, -0.21, -0.24, -0.22
**Average**	**0.176 ± 0.011**	**-0.233 ± 0.022**

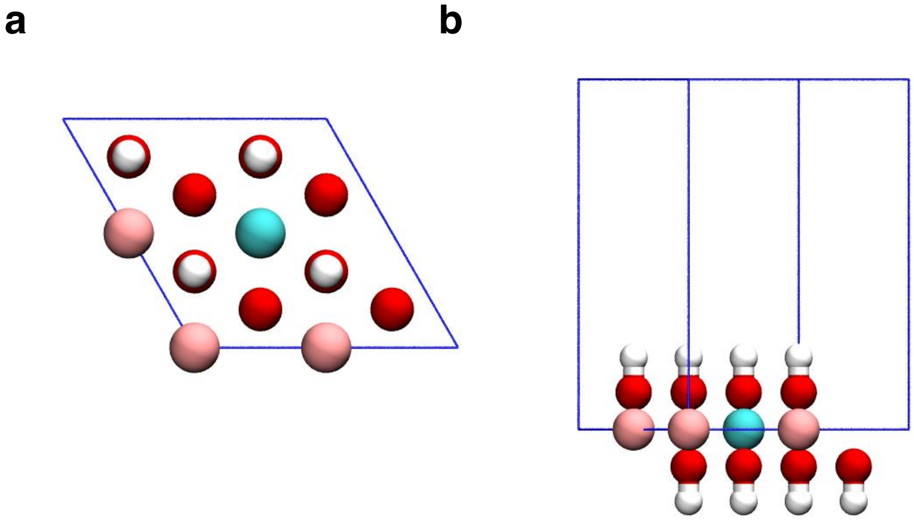

Supplementary Figure 1: Unit cell of LDH

Layered double hydroxide unit cell **(a)** top and **(b)** side view. Colours are: Al – cyan, Mg – pink, O – red and H – white.

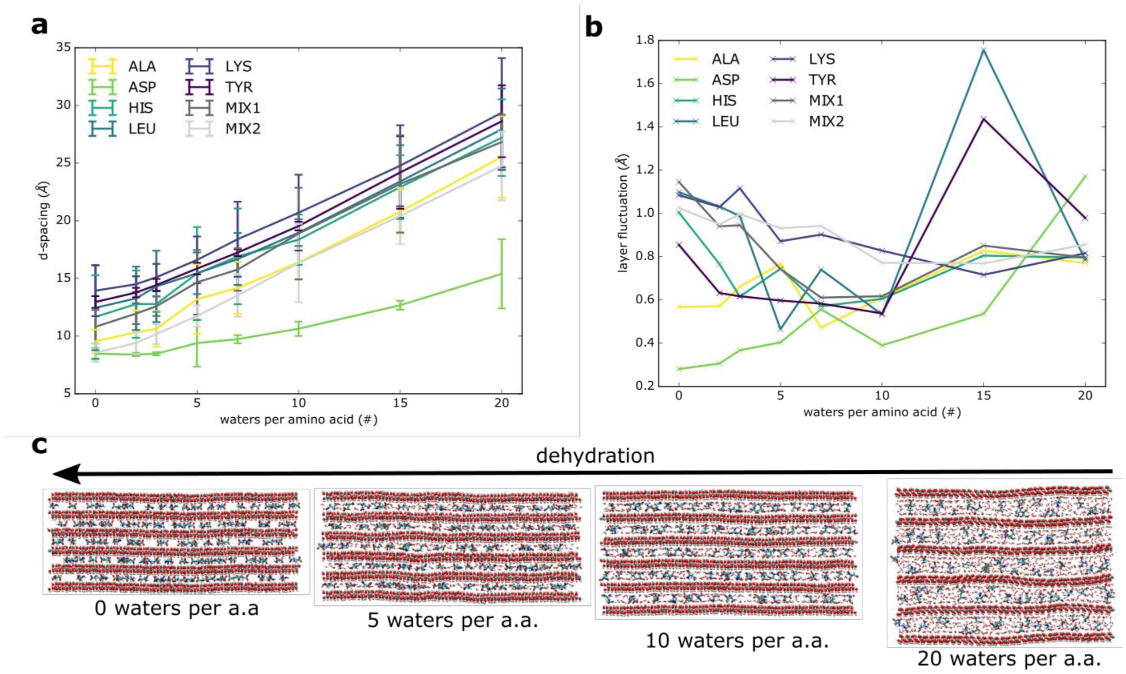

Supplementary Figure 2: *d*-spacing and layer undulations

(**a**) LDH interlayer *d*-spacing as a function of hydration (waters per amino acid), with different intercalated with amino acids. Note: ASP is doubly charged, so is present in half the amount with respect to other amino acids, MIX2 has 40% of amino acid load with 60% of Cl^- counterbalancing ions). (**b**) LDH layer undulations as a function of hydration (waters per amino acid). (**c**) snapshots of the dehydration of ASP-intercalated LDH.

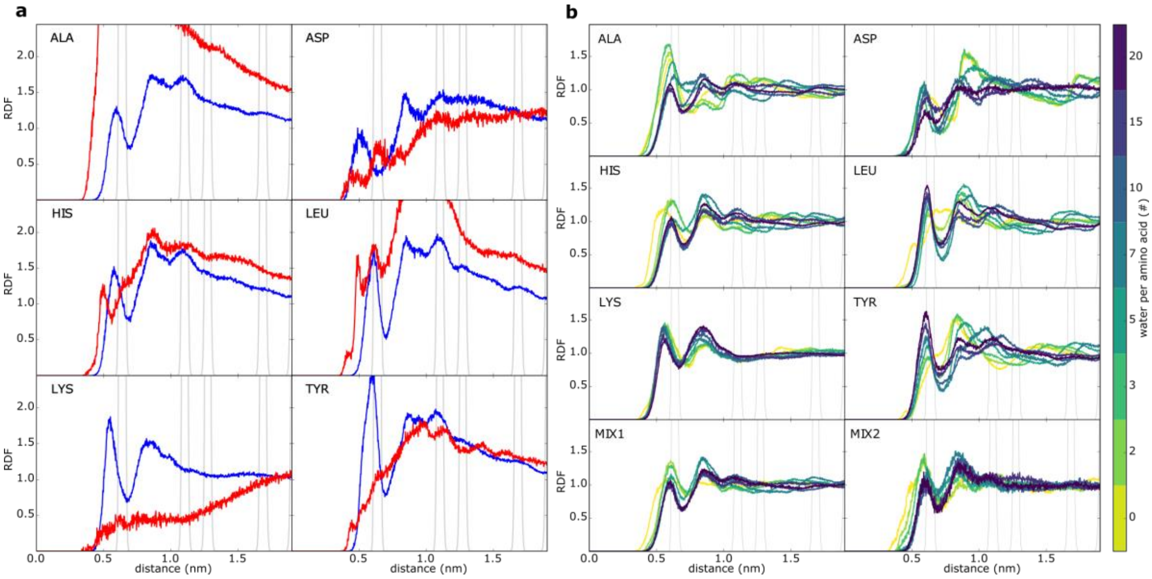

Supplementary Figure 3: Radial Distribution Function of amino acids

We report the Radial Distribution Function (RDF) of amino acids and peptides C-termini with respect of LDH aluminium atoms. **(a)** RDF of homo-peptides. Blue – dimer, red – hexamer. **(b)** RDF of amino acids, with colour gradient as a function of hydration. In grey, the distribution of aluminium atoms is indicated. A clear first shell is present (0.64 nm), indicating that the C-terminal of amino acids arrange with the same repetition of LDH unit cells. The second peak (0.8 nm) corresponds to C-termini across the LDH layer. The following peaks (1.11 nm and 1.28 nm) correspond to C-termini arranged on the diagonal unit cell and their neighbouring ones. The LDH templating effect on amino acids arrangement is observed for all adsorbed amino acids. Aspartate features a slightly different pattern, as one aspartate holds a 2– charge and therefore is shared between two unit cells of LDH, often interacting *via* both its carboxylic groups. Amino acid mixtures (MIX1 and MIX2) show the same behaviour as the pure systems.

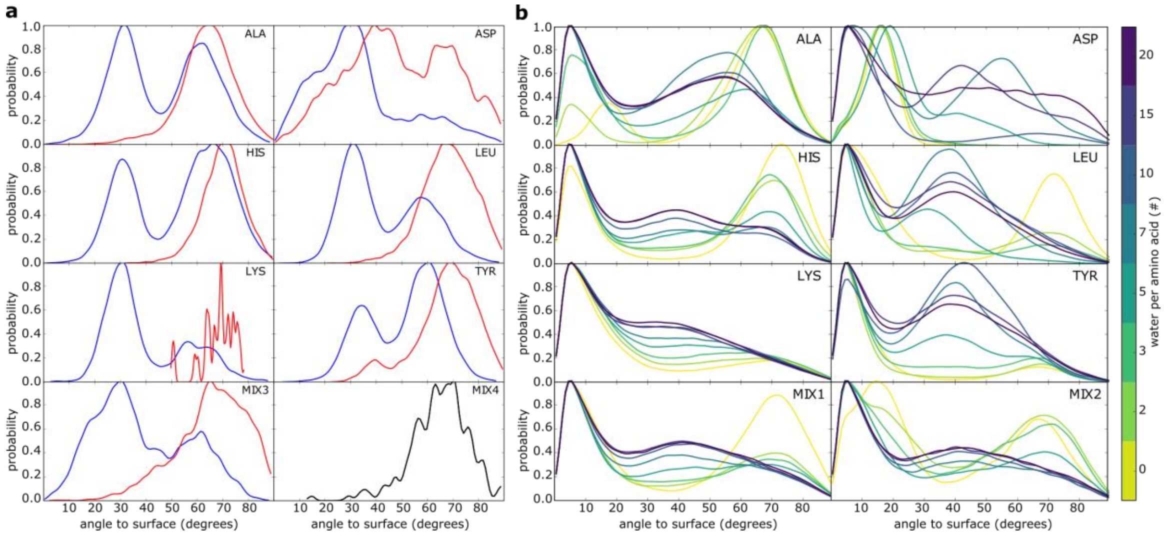

Supplementary Figure 4: Alignment of adsorbed species with respect to LDH

A vector is assigned between C and N atoms of the adsorbed amino acid's backbone, and its elevation (in spherical coordinates) reported. A 0 degrees elevation corresponds to a backbone perpendicular to the LDH surface, a 90 degrees to a backbone parallel to it. **(a)** Alignment of homo-peptides (top three rows) and hetero-peptides (bottom row) with respect to the surface. Dimers, hexamers and 24mers are shown in blue, red and black, respectively. The C-termini of the shortest peptides align like those of single hydrated amino acids; while in the case of longer peptides (including 24mer, with an exception of hexa-aspartate) C-termini align near-perpendicularly to the surface. Polyaspartates can co-adsorb with their side-chains and so remain parallel to the surface. **(b)** Alignment of single amino acids, as a function of their hydration level. At high levels of hydration, amino acids mainly adsorb by their C-terminals. Although still mobile, the backbones show preferential alignments: either planar, or at 40 degree to the surface. Upon dehydration, backbones arrange more uniformly, either perpendicular or parallel to the surface. This is because C-termini have two oxygens, able either to adsorb to the same LDH surface, or to bridge to the opposing one. Due to the specific adsorption behaviour of lysine and aspartate, the alignments to the surface do not follow the general trend. Tyrosine favours a planar alignment upon dehydration, due to π-stacking of the aromatic rings of the side chains.

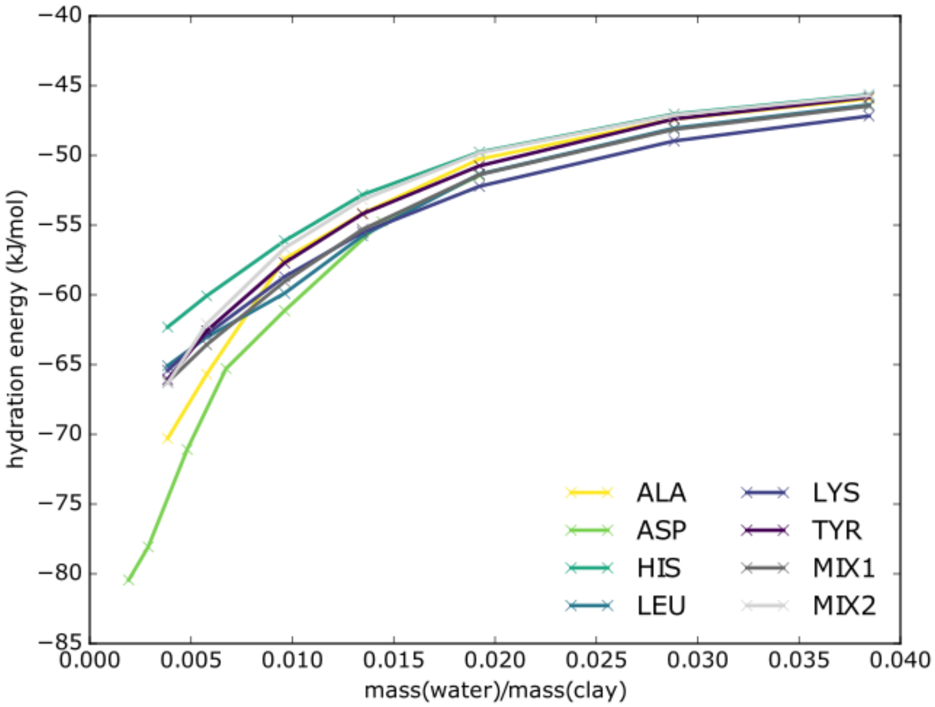

Supplementary Figure 5: Hydration energy

The energies associated with dehydration of the LDH-amino acid interlayer, as a function of intercalated amino acid composition. The hydration energy of a pure water system is -33.25 kJ mol-1. All of the systems are below this value, and therefore will rehydrate. Notably, the more dehydrated is the system; the more rehydration is energetically favourable.

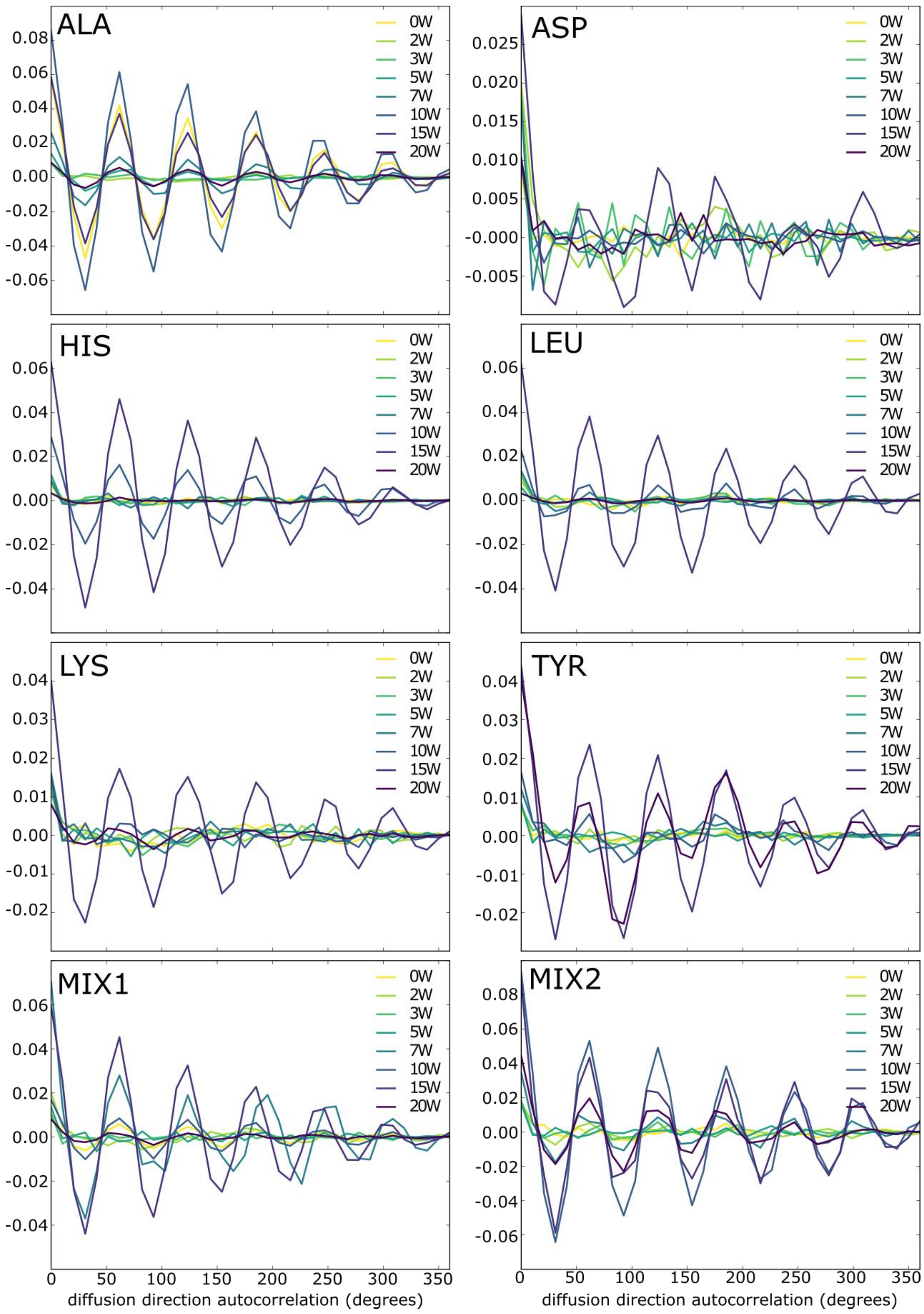

Supplementary Figure 6. Autocorrelation of amino acid diffusion direction on LDH surface

We describe the diffusion of each amino acid adsorbed on the LDH surface as a two dimensional vector, i.e. a direction on the *xy*-plane. In each system, six distinct peaks are observed, with higher intensities observed in more hydrated systems. This indicates that amino acids are more mobile in hydrated systems, with a strong hexagonal movement preference templated by LDH metal ions arrangement (see Supplementary Fig. 1).

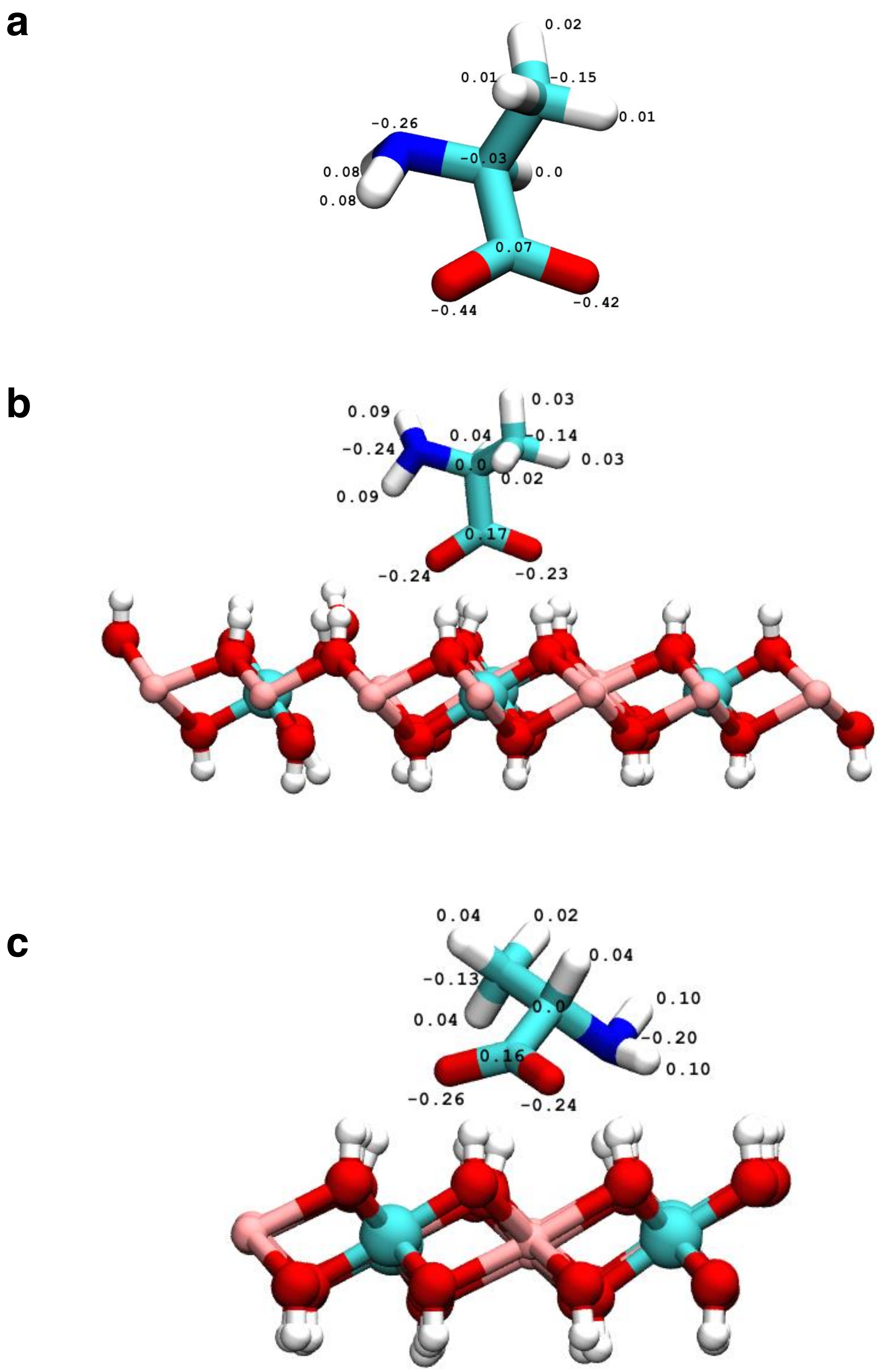

Supplementary Figure 7: Partial charges on alanine

(a) Atomic charges calculated on alanine in vacuum. **(b)** Atomic charges of an alanine adsorbed on the LDH surface via its C-terminal **(c)** Atomic charges an alanine adsorbed via both its C- and N-terminal.

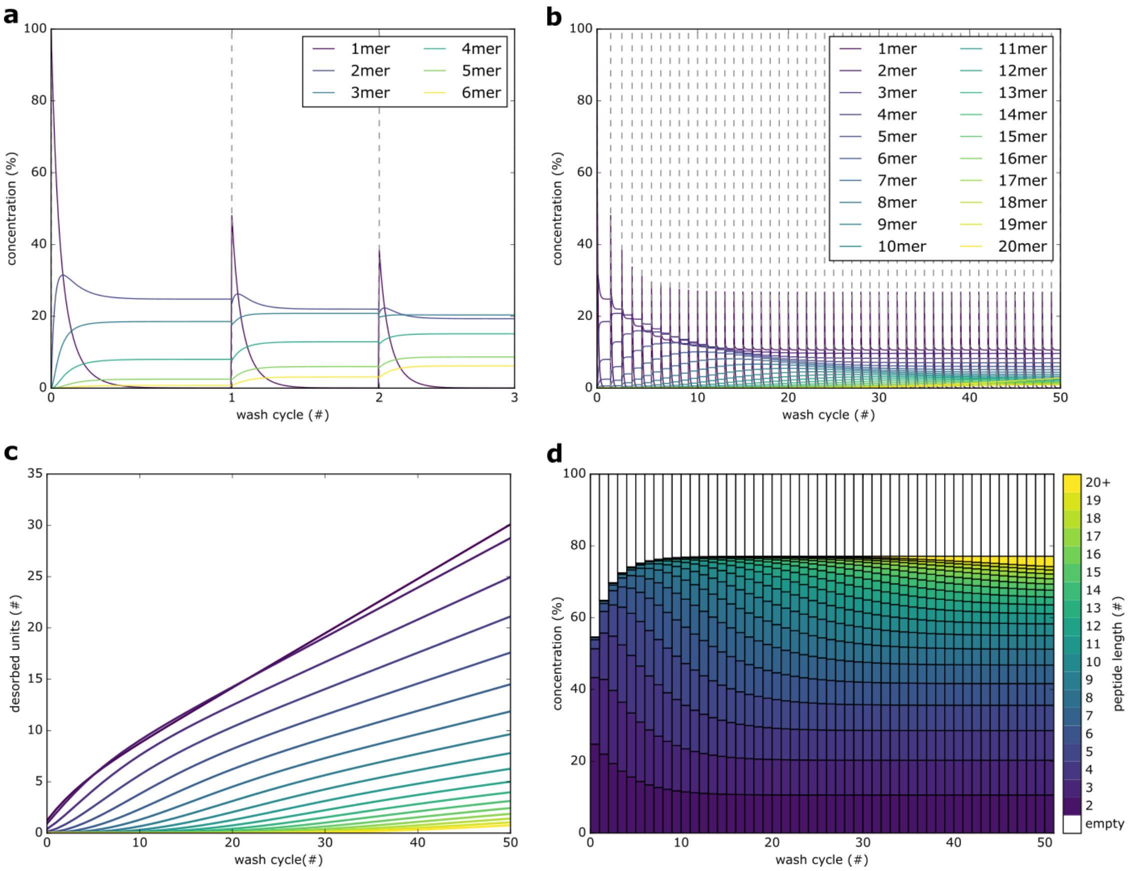

Supplementary Figure 8: Kinetic model of amino acids polymerisation on LDH
(a) Peptide concentration in an LDH interlayer per wash cycle. Three wash cycles are shown. At the beginning of each cycle, the system is saturated with monomers that subsequently react with existing multimeric species, until being totally consumed. At the end of each cycle, 5% of each species is removed from the system, to simulate species release upon LDH rehydration. **(b)** After a 50 wash cycles, higher order oligomers become detectable. **(c)** Cumulant of all species released from LDH, per wash cycle. After a short equilibration phase, linear trends can be observed for each specie, showing that LDH undergoing dehydration-rehydration cycles should produce a steady amount of peptides of different stoichiometries. **(d)** Species concentration upon each cycle convergence. Upon convergence, LDH is always >20% empty, and is thus capable of adsorbing new monomeric amino acids during the next rehydration phase.

Supplementary References

1. King, J. L. & Jukes, T. H. Non-Darwinian evolution. *Science* **164,** 788–798 (1969).
2. Degiacomi, M., Erastova, V. & Wilson, M. Easy creation of polymeric systems for molecular dynamics with Assemble! *Phys*. *Commun*. **202**, 304-309 (2016).
